# Cost-Effectiveness of Computer-Assisted Cognitive Behavioral Therapy for Depression Among Adults in Primary Care

**DOI:** 10.1001/jamanetworkopen.2024.44599

**Published:** 2024-11-14

**Authors:** Shehzad Ali, Feben W. Alemu, Jesse Owen, Tracy D. Eells, Becky Antle, John Tayu Lee, Jesse H. Wright

**Affiliations:** 1 Department of Epidemiology and Biostatistics, Schulich School of Medicine, Western University, London, Ontario, Canada; 2 Mental Health and Addictions Research Group, Department of Health Sciences, University of York, York, United Kingdom; 3 Department of Psychology, Macquarie University, Sydney, Australia; 4 Department of Counseling Psychology, University of Denver, Denver, Colorado; 5 Department of Psychiatry and Behavioral Sciences, University of Louisville School of Medicine, Louisville, Kentucky; 6 Kent School of Social Work, University of Louisville, Louisville, Kentucky; 7 Institute of Health Policy and Management, National Taiwan University, Taipei, Taiwan

## Abstract

**Importance:**

Approximately 1 in 5 adults are diagnosed with depression in their lifetime. However, less than half receive help from a health professional, with the treatment gap being worse for individuals with socioeconomic disadvantage. Computer-assisted cognitive behavioral therapy (CCBT) is an effective and convenient strategy to treat depression; however, its cost-effectiveness in a sociodemographically diverse population remains unknown.

**Objective:**

To evaluate the cost-effectiveness of clinician-supported CCBT compared with treatment as usual (TAU) in a primary care population with a substantial number of patients with low income, limited computer or internet access, and lack of college education.

**Design, Setting, and Participants:**

This economic evaluation was a randomized clinical trial–based cost-effectiveness analysis. The trial was conducted at the Departments of Family and Geriatric Medicine and Internal Medicine at the University of Louisville. Enrollment occurred from June 24, 2016, to May 13, 2019. Participants had mild to moderate depression and were followed up for 6 months after treatment completion. The last follow-up assessment was conducted on January 30, 2020. Statistical analysis was performed from August 2023 to August 2024.

**Exposure:**

CCBT intervention was provided for 12 weeks and included 9 modules ranging from behavioral activation and cognitive restructuring to relapse prevention strategies, supported by telephonic sessions with a clinician, in addition to TAU, which included standard clinical management in primary care.

**Main Outcomes and Measures:**

The primary health outcome was quality-adjusted life years (QALYs), estimated using the Short-Form 12 questionnaire (SF-12). The secondary outcome was treatment response, defined as at least 50% improvement in the Patient Health Questionnaire. The intervention cost included sessions with mental health clinicians and the cost of the CCBT software, plus the cost of loaner computer and internet data plan for low-resource households. An incremental cost-effectiveness ratio (ICER) was computed, while adjusting for baseline scores, age, and sex. The cost-effectiveness acceptability curve presented the probability of CCBT being cost-effective for a range of willingness-to-pay values.

**Results:**

Among the 175 primary care patients included in this study, 148 (84.5%) were female; 48 (27.4%) were African American, 2 (1.2%) were American Indian or Alaska Native, 4 (2.5%) were Hispanic, 106 (60.5%) were White, and 15 (8.6%) were multiracial; and the mean (SD) age was 47.03 (13.15) years. CCBT was associated with better quality of life and higher chance of treatment response at the posttreatment and 6-month time points, compared with the TAU group. The ICER for CCBT was $37 295 (95% CI, $22 724-$66 546) per QALY, with a probability of 89.4% of being cost-effective at a willingness-to-pay threshold of $50 000/QALY. The ICER per case of treatment response was $3623 (95% CI, $2617-$5377).

**Conclusions and Relevance:**

In this trial-based economic evaluation, CCBT was found to be cost-effective, compared with TAU, in primary care patients with depression. As this study included individuals with low income and with limited internet access who are underrepresented in cost-effectiveness studies, it has important policy implications for addressing unmet needs in sociodemographically diverse populations.

## Key Points

**Question:**

Is computer-assisted cognitive behavioral therapy (CCBT) plus treatment as usual, compared with treatment as usual alone, cost-effective for treating depression in primary care?

**Findings:**

In this economic evaluation of a socioeconomically diverse population of 175 patients with mild to moderate depression, CCBT had higher quality-adjusted life years (QALYs) and treatment success at a marginal cost. The incremental cost-effectiveness ratios were $37 295 per QALY and $3623 per case of treatment success.

**Meaning:**

These findings suggest that CCBT is a cost-effective strategy for treating depression in a diverse primary care population.

## Teaser

This economic evaluation assesses the cost-effectiveness of clinician-supported computer-assisted cognitive behavioral therapy (CBT) vs treatment as usual in a primary care population with a substantial number of patients with low income, limited computer or internet access, and lack of college education.

## Introduction

Depression is a common mental disorder with a lifetime prevalence of 18.4% in the adult population in US.^1^ In 2020, approximately 1 in every 10 adults reported being depressed in the past year,^2^ with a health impact of 807.2 disability-adjusted life years (DALYs) per 100 000 population.^3^ Moreover, the burden of depression is distributed inequitably, with highest prevalence in those with lowest household income.^2^ This health burden places substantial economic strain on the US health care system and the overall society, with an estimated annual cost of $382.4 billion.^2,4^

Psychological therapies, particularly cognitive behavioral therapy (CBT), have been found to be effective in the management of depression^5,6,7,8,9^. However, a treatment gap for depression remains a major policy concern.^2^ Estimates suggest that fewer than half of all patients with depression report receiving help from a medical professional.^10^ The gap is particularly worse for the socially marginalized population groups, including those with low levels of income and/or education.^2,11,12,13^ An important contributing factor is the high cost of psychotherapy, which poses a substantial barrier to care-seeking.^14^ Other challenges are the limited availability of trained psychotherapists, long wait times, travel distance, and the stigma associated with in-person mental health interventions.^15,16^

Accessible treatment options for depression are needed to reduce population health burden and financial impact on the health care system. Technological developments have led to the delivery of CBT using computer programs that facilitate remote access to therapy. Evidence suggests that computer-assisted CBT (CCBT), provided as a standalone treatment or in combination with treatment as usual (TAU), is an effective strategy, with effect size varying across studies, depending partly on whether CCBT was supported by a clinician.^9,17,18,19^ Moreover, studies have found that CCBT may be cost-effective compared with control conditions, although the evidence is heterogeneous, both in terms of methods and setting, making it challenging to draw generalizable conclusions.^20,21,22,23^

One limitation of CCBT studies is the tendency to overweight samples with well-educated individuals with computer skills and internet access^19,24^; and the effectiveness of CCBT in socioeconomically marginalized populations, including individuals with limited access to the internet, has received much less attention.^14^ To address this limitation, we previously conducted a randomized clinical trial (RCT) to evaluate the effectiveness of CCBT for depression compared with treatment as usual (TAU) in primary care, with substantial representation of patients without computer or internet access, and those with low income and lack of college education.^25^ Our study found that after 12 weeks of treatment, CCBT improved patient outcomes, which were maintained at the 6-month follow-up.^25^ Using data from the same RCT, the aim of the present study is to evaluate the within-trial cost-effectiveness of CCBT for depression, compared with TAU, in the primary care setting.

## Methods

### Study Design

The primary RCT used for the cost-effectiveness analysis has been published elsewhere^25^; it recruited 175 participants from the clinical practices of the Departments of Family and Geriatric Medicine and Internal Medicine at the University of Louisville. Enrollment occurred from June 24, 2016, to May 13, 2019. The last follow-up assessment was conducted on January 30, 2020. Adults with a Patient Health Questionnaire (PHQ-9) score of 10 or above, a cutoff conventionally used to screen for depression,^26^ were included in the trial and randomized to either the CCBT group or TAU. All participants provided written informed consent, which covered the use of their deidentified data for future research purposes, including secondary analyses. The RCT was approved and monitored by the University of Louisville institutional review board. The CCBT participants received, in addition to TAU, the 9-lesson Good Days Ahead (GDA) computer program over 12 weeks and as many as 12 telephonic sessions with a mental health clinician. TAU included the standard clinical management in primary care practices and was not controlled.

This economic evaluation’s cost-effectiveness analysis was conducted from the US health care payer perspective. The time horizon of the analysis was aligned with the trial follow-up period from baseline to 6-months postintervention. Two health outcomes were used separately for the cost-effectiveness analysis: (1) the quality-adjusted life year (QALY), commonly used for resource allocation decisions; and (2) treatment response, which is relevant to clinical decision-making.

### Costs and Outcomes

QALYs were estimated using the Short-Form 12 questionnaire (SF-12) version 2, which is a standard tool used in economic evaluation studies.^27,28,29^ SF-12 has been validated across populations and health conditions, including individuals with mental health conditions.^30,31,32,33,34^ In patients with depression, SF-12, particularly the mental component score (MCS), has been shown to be highly reliable and valid as a diagnostic and monitoring tool, with an area-under-the-curve (AUC) statistic between 0.85 and 0.92.^35,36,37^ The questionnaire asks respondents about their health in the past 4 weeks on the following 8 domains: general health, physical functioning, physical role, emotional role, social functioning, bodily pain, vitality, and mental health. Responses are summarized in terms of physical component score (PCS) and MCS, each ranging from 0 to 100, with higher score implying better health.^28,32^ These scores are norm-based, standardized with a mean of 50 and a standard deviation of 10, allowing for comparison with the general population.^27^ SF-12 responses were converted into utility (ie, health-related quality of life) values using US population-specific tariff.^28^ Utility values range between 0 and 1, with 1 implying perfect health and 0 implying death.^38^ Utility data were used to calculate QALYs over the study period using the AUC approach.^39^ The regression models, described later, adjusted for the baseline utility derived using the SF-12 response (range: 0-1).

The treatment response outcome was based on the 9-item PHQ-9, which is a common measure of depression in clinical practice. The validity and reliability of PHQ-9 has been demonstrated in the US population.^26^ Each item of the PHQ-9 scale is rated on a 4-point Likert scale, yielding a total score between 0 and 27.^26^ A reduction of at least 50% in PHQ-9 score has been proposed to define successful response to treatment.^40^ This approach does not favor higher or lower baseline depression severity, which is a criticism leveled against defining success in terms of absolute improvement in the PHQ-9 score; moreover, it is specific to each patient and clinically meaningful.

The intervention cost was calculated using individual-level data on the number of sessions attended by each participant in the CCBT group and multiplying this by the Medicare reimbursement rate for a psychotherapy session delivered by a licensed clinical social worker ($71.10 per session).^41^ Additionally, we accounted for the cost of the GDA software ($75).^42^ CCBT participants were offered the option to borrow a laptop (ie, Chromebook, cost of $253 per unit)^43^ and a MiFi device with a data plan for 3 months (at $20.1 per month).^44^ The cost of these devices was covered by the program. Out of 95 participants in the CCBT group, 17 borrowed a laptop while others used their own devices and data plans; 9 of the 17 borrowed laptops were returned. The analysis incorporated the full cost for lost laptops, while returned laptops incurred a 30% depreciation adjustment. Costs were measured in 2021 US dollars.

### Statistical Analysis

The missingness pattern in the data was assumed to be missing at random (MAR). Multiple imputation (MI) by chained equations (MICE) method was used to handle missingness.^45,46^ In particular, we used MICE with predictive mean matching (PMM) from the 5 *k*-nearest neighbors. PMM imputes observations based on observed values, preserving the distribution of the data. MI is a robust approach for handling missing data, violations to normality, and sampling variation, and has reasonable performance and computational time compared with other resampling approaches, including MI bootstrap.^47^

We ran MI for 1000 iterations, generating 1000 datasets. Using the multiply imputed datasets, we conducted regression analysis using generalized linear models (GLMs); the treatment response model used a binomial distribution and a logit link, and cost and QALY models used gaussian distribution and an identity link. The models were estimated jointly to allow for correlation between costs and health outcomes, assuming that the residuals follow a gaussian distribution and the random effects are correlated. To account for baseline differences between groups, we controlled for baseline utility in the QALY regression,^48^ and baseline PHQ-9 score in the treatment response regression, in addition to adjusting for age and sex. The coefficient on the CCBT variable represented the difference in costs and outcomes between CCBT and TAU groups. Rubin rule was used to pool regression estimates from the multiply imputed datasets, to compute the overall estimates, standard errors, and confidence intervals around coefficients.

Next, an incremental cost-effectiveness ratio (ICER) was computed as a ratio of the coefficient on the CCBT variable in the cost and QALY regressions. The ICER value represents the incremental cost of the CCBT intervention, compared with TAU, for a gain of 1 additional QALY.

Uncertainty in cost-effectiveness decision was investigated using the cost-effectiveness acceptability curve (CEAC), which represents the probability of CCBT being cost-effective for a range of values that a decision-maker may be willing to pay for a health gain of 1 QALY.^49^ To investigate longer-term cost-effectiveness of CCBT beyond the trial, we conducted extrapolation analyses across different time horizons, following the CCBT intervention: 12, 24, 36, 48, and 60 months. For each time horizon, 2 scenarios were investigated in relation to the durability of treatment benefit beyond the observed trial period: (1) treatment benefit reduces linearly over time, with utility values reaching pretreatment levels at the end of the time horizon; and (2) treatment benefit remains stable until the end of the time horizon. For the cost analysis, no additional intervention cost was incurred beyond the trial period, and we made the conservative assumption that the CCBT group did not reduce health service use.

All analyses were performed from August 2023 to August 2024 in R Statistical Software version 4.4.0 (R Project for Statistical Computing).^50^ Two-tailed tests were used at a .05 level of significance. We used *VIM* and *naniar* packages to visualize and determine the missingness mechanism; the *mice* package to run the multiple imputations; the *doRNG* package for parallelization of the imputations; the *glmmTMB* package for joint estimation of models; the *ggplot2* and *ggpubr* packages to create the figures; and the *dplyr* package for general data manipulation.^51,52,53,54,55,56,57,58^

## Results

Descriptive statistics of study participants are presented elsewhere.^25^ Briefly, the sample of 175 primary care patients was predominantly female (148 [84.5%]), with a mean age (SD) of 47.03 (13.15) years. The sample included a substantial representation of racial and ethnic groups (48 African American [27.2%], 2 American Indian or Alaska Native [1.2%], 4 Hispanic [2.5%], and 15 multiracial [8.6%]), while the remaining 106 (60.5%) identified as White. Respondents, totaling 74.6%, reported having less than a college education and 61.5% reported an annual income of less than $30 000. Baseline characteristics were similar across the treatment groups, with small differences. For instance, a majority of participants in both groups were White (CCBT: 61.2%; TAU: 59.7%), with African Americans representing a substantial proportion in both groups (CCBT: 25.9%; TAU: 28.6%). There were small differences between groups in the proportion of people with annual income of $0 to 14 999 (CCBT: 48.6%; TAU: 40.8%) and the proportion of women (TAU: 88.8%; CCBT: 80.9%). A comparable proportion of participants were currently receiving antidepressant treatment (CCBT: 23.4%; TAU: 24.1%).

**Figure 1.**
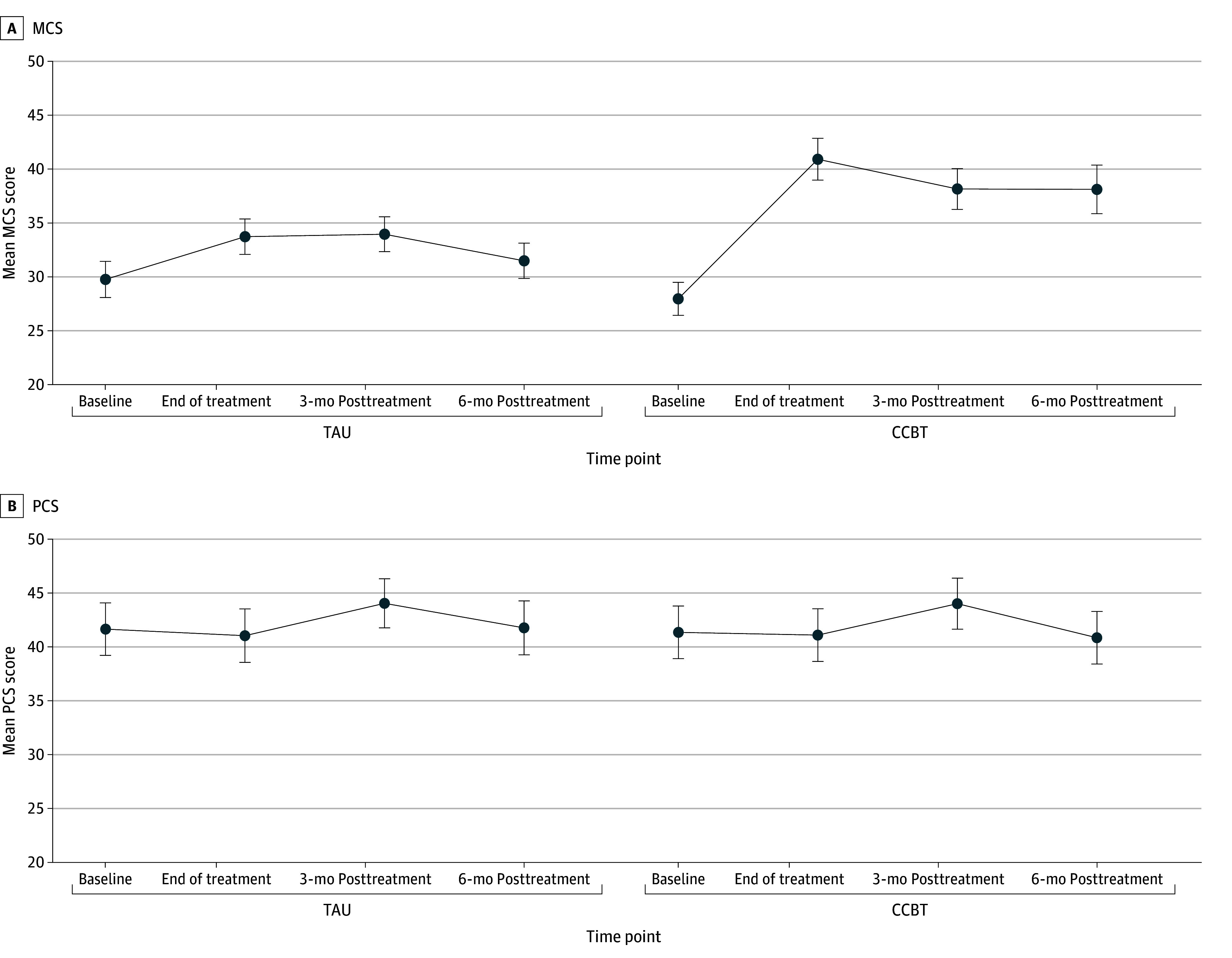
Computer-Assisted Cognitive Behavioral Therapy (CCBT) vs Treatment-as-Usual (TAU) Groups on Short-Form 12 Questionnaire Mental Component Score (MCS) and Physical Component Score (PCS)

Figure 1 shows changes in SF-12 MCS and PCS scores from the study baseline through the follow-up period. The CCBT participants started at a mean score on the MCS scale of 27.96 (95% CI, 26.42-29.49), as compared with the TAU group mean of 29.76 (95% CI, 28.08-31.44); the difference between groups was not statistically significant. In the CCBT group, the mean MCS score improved significantly by the end of treatment (40.91 [95% CI, 38.97-42.85]) compared with a smaller increase in the TAU group (33.73 [95% CI, 32.09-35.37]). In the following period, MCS scores dropped slightly in both groups; the CCBT group had a higher mean of 38.12 (95% CI, 35.86-40.37) at the end of follow-up (ie, 6 months) compared with 31.49 (95% CI, 29.85-33.12) in the TAU group. The PCS scores in the 2 groups remained stable during the follow-up.

The SF-12 scores were converted into utility (quality of life) values (Table 1). The utility values were higher in the CCBT group, compared with TAU, at all follow-up time points and were statistically significant at 12-week and 6-month time points after adjusting for baseline utility. Treatment success, defined as at least 50% improvement in PHQ-9 score, was observed in 58.21% (95% CI, 45.54%-69.94%) and 33.96% (95% CI, 21.89%-48.36%) of patients at 12-week time point in the CCBT and TAU groups, respectively (ie, patients receiving CCBT had an approximately 71% higher treatment success rate than TAU). The differences remained statistically significant at 3-month and 6-month follow-up, with CCBT patients achieving 73.3% and 80.6% higher treatment success, respectively, compared with TAU.

**Table 1.** Quality-of-Life Scores and Percentage of Cases With Treatment Success

Time point	Quality of life, utility value (95% CI)^a^			Treatment success, % of patients (95% CI)^b^		
	CCBT	TAU	*P* value	CCBT	TAU	*P* value
Baseline	0.489 (0.466-0.511)	0.495 (0.468-0.522)	.75	[Reference]	[Reference]	
Posttreatment (12 wk)	0.619 (0.590-0.656)	0.563 (0.535-0.598)	.01	58.21 (45.54-69.94)	33.96 (21.89-48.36)	.008
3-mo Follow-up	0.601 (0.562-0.641)	0.606 (0.573-0.640)	.62	50.00 (37.74-62.27)	28.85 (17.55-43.27)	.02
6-mo Follow-up	0.608 (0.563-0.653)	0.561 (0.525-0.597)	.05	45.16 (32.68-58.24)	25.00 (13.70-40.65)	.03

Abbreviations: CCBT, computer-assisted cognitive behavioral therapy; TAU, treatment as usual.

^a^ Values at follow-up time points were adjusted for baseline utility.

^b^ Success defined as an improvement in Patient Health Questionnaire–9 of ≥50% at follow-up.

In terms of the cost analysis, the CCBT participants attended a mean (IQR) of 8.4 (7-11) sessions and received a mean (IQR) of 3.5 (3.2-4.4) hours of therapy. The computing cost of a laptop and MiFi plan was incurred by 17 of 95 participants; this was averaged over the CCBT group to arrive at a mean cost of $39.3 per participant. The overall (unadjusted) mean cost of CCBT per participant, compared with TAU, was $709.6 (95% CI, $708.1-$711.1).

Table 2 (and eTable 1 in Supplement 1) present the results of regression analyses for QALYs, RSCI and costs, controlling for baseline health, age and sex. In the QALY analysis, the CCBT intervention was associated with a statistically significant improvement of 0.021 (95% CI, 0.003-0.039), compared with TAU, over the study period. Higher baseline utility level, estimated using the SF-12 questionnaire, was associated with higher QALYs (0.548 [95% CI, 0.470-0.625]). In the treatment response analysis, CCBT was associated with a statistically significant odds ratio of 2.83 (95% CI, 1.33-6.05) for improvement of at least 50% on the PHQ-9 score at 12 weeks after treatment. The odds ratio at 6-month time point was 2.53 (95% CI, 1.11-5.78) and statistically significant. Age and sex associations were not statistically significant in either analysis. For the cost regression, the incremental cost for CCBT, compared with TAU, was $714.6 (95% CI, $662.5-$766.7).

**Table 2.** Regression Analyses of Cost, QALYs, and Treatment Response, Adjusting for Age, Sex, and Baseline Utility

Variables	Cost	*P* value	QALY	*P* value
	Coefficient (95% CI) [SE]		Coefficient (95% CI) [SE]	
CCBT = 1	714.64 (662.53 to 766.75) [26.4]	<.01	0.0209 (0.003 to 0.039) [0.009]	.02
Age	−0.67 (−2.67 to 1.32) [1.01]	.51	0.0002 (−0.001 to 0.001) [0.0003]	.60
Female	43.35 (−32.47 to 119.17) [38.4]	.26	−0.00624 (−0.032 to 0.020) [0.013]	.63
SF-12 baseline utility	195.80 (−35.93 to 427.52) [117.3]	.10	0.548 (0.470 to 0.625) [0.039]	<.01
(Intercept)	−104.102 (−276.48 to 68.28) [87.3]	.24	0.138 (0.080 to 0.197) [0.030]	<.01
	**12-wk Response**		**6-mo Response**	
	**OR (95% CI) [SE]**	***P* value**	**OR (95% CI) [SE]**	***P* value**
CCBT	2.834 (1.327 to 6.054) [0.383]	.008	2.531 (1.107 to 5.783) [0.417]	.03
Age	1.018 (0.990 to 1.046) [0.014]	.21	0.989 (0.958 to 1.020) [0.016]	.47
Sex	1.414 (0.497 to 4.027) [0.528]	.51	0.7257 (0.2539 to 2.074) [0.530]	.55
Baseline PHQ-9 score	1.007 (0.949 to 1.070) [0.030]	.81	0.9997 (0.9383 to 1.065) [0.030]	.99
(Intercept)	0.140 (0.022 to 0.887) [0.933]	.04	0.740 (0.106 to 5.151) [0.980]	.76

Abbreviations: CCBT, computer-assisted cognitive behavioral therapy; OR, odds ratio; PHQ-9, 9-item Patient Health Questionnaire; SF-12, Short-Form 12 questionnaire; QALY, quality-adjusted life year.

**Figure 2.**
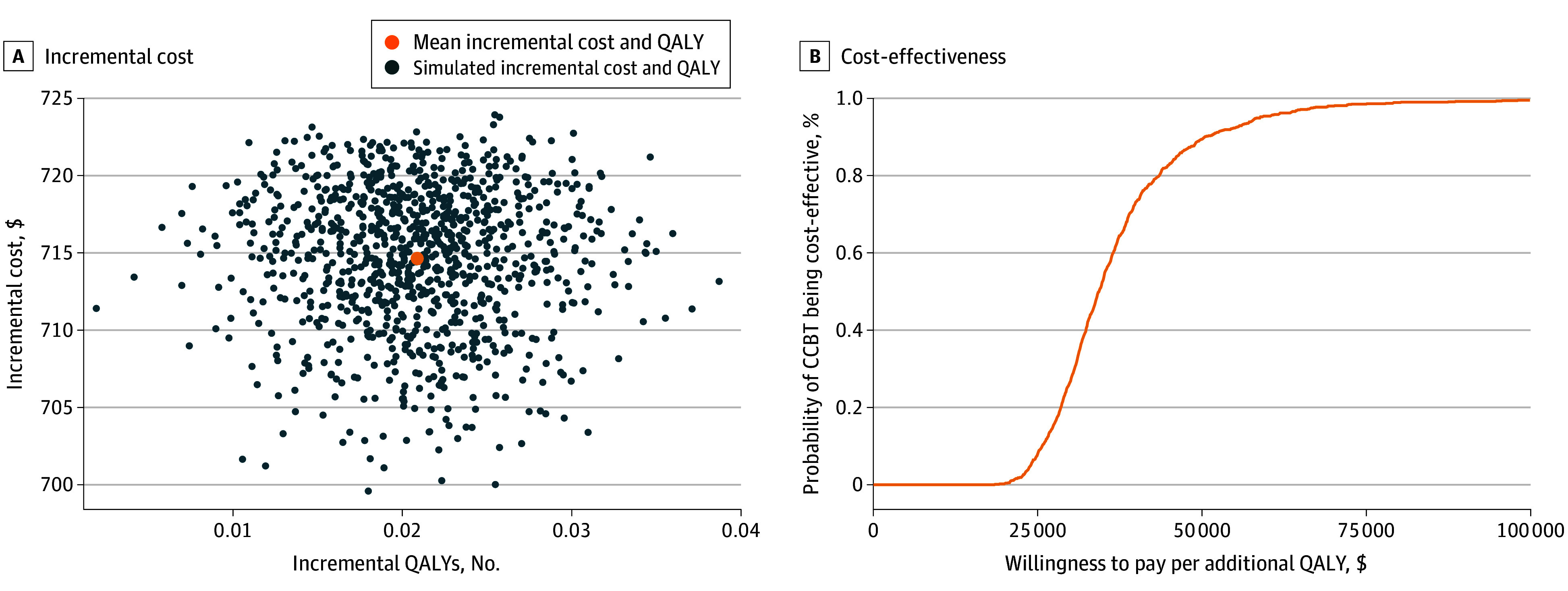
Cost-Effectiveness Plane and Acceptability Curve Based on Incremental Costs and Quality-Adjusted Life Years (QALYs) The cost-effectiveness plane in the figure presents the joint distribution of incremental costs and QALYs for cost of computer-assisted cognitive behavioral therapy (CCBT) compared with treatment as usual, based on 1000 simulations.

To inform the health care decision-making under uncertainty of evidence, the cost-effectiveness plane in Figure 2A presents the joint distribution of incremental costs (y-axis) and QALYs (x-axis) for CCBT compared with TAU, based on 1000 simulations. The figure shows that while CCBT is more expensive than TAU, it is also more effective in terms of QALY gains. The ICER value (ie, the incremental cost) of CCBT per QALY gain is $37 295 (95% CI, $22 724-$66 546) (Figure 2A). Figure 2B presents the CEAC plot which represents the probability of CCBT being cost-effective (y-axis) for a range of willingness-to-pay (WTP) values per QALY (x-axis) that may be used by health system decision-makers in the US. The probability of CCBT being cost-effective at a WTP of $50 000/QALY was 89.4%, which increases to 99.5% for WTP threshold of $100 000/QALY.

The extrapolation analysis based on longer time horizon found that, as the time horizon increases, the ICER values decrease (eTable 2 in Supplement 1). ICER values are lower in scenario 2, which assumed that the treatment benefit remains stable until the end of the time horizon compared with scenario 1, which assumed waning of treatment effect over time. Across all scenarios, the ICER values were below the WTP threshold values.

**Figure 3.**
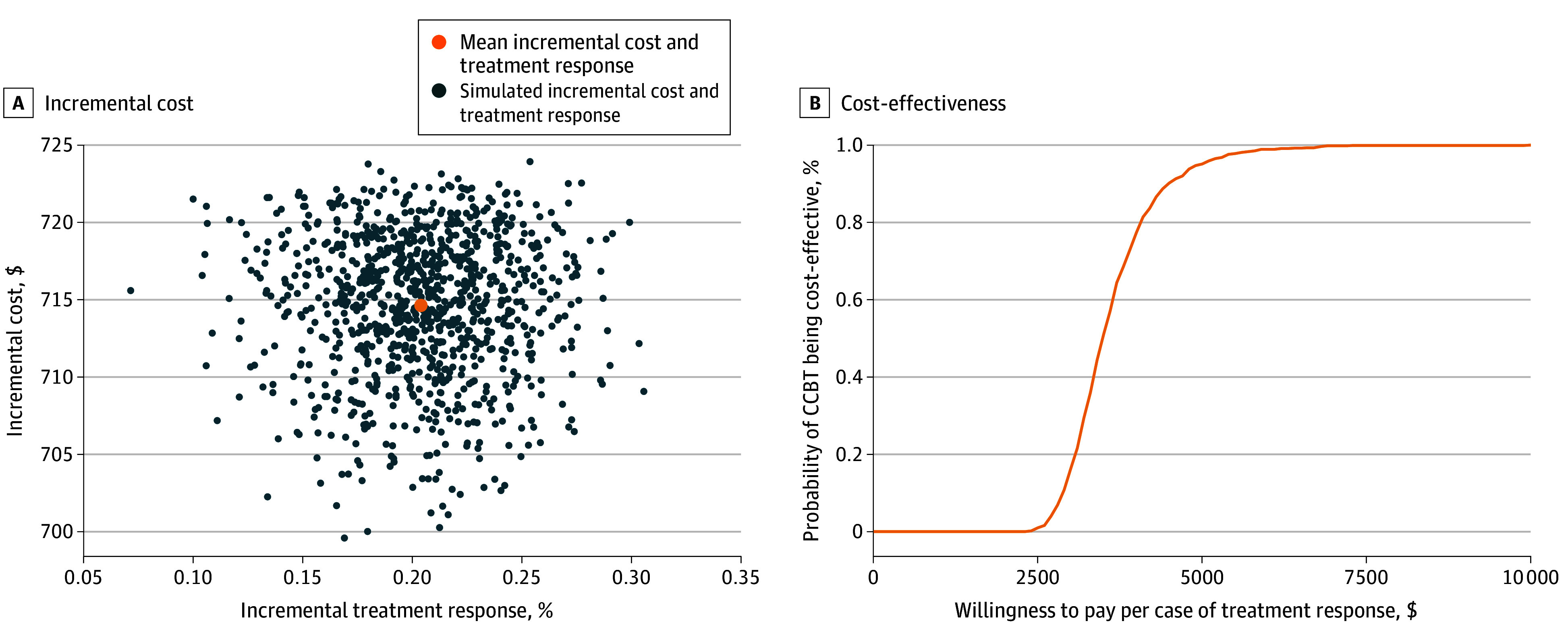
Cost-Effectiveness Plane and Acceptability Curve Based on Incremental Costs and Cases With Successful Treatment Response The figure presents the joint distribution of the incremental probability of treatment response and the incremental cost of computer-assisted cognitive behavioral therapy (CCBT) compared with treatment as usual, based on 1000 simulations.

Finally, Figure 3A presents the joint distribution of the incremental probability of treatment response and the incremental cost of CCBT compared with TAU. The figure shows that for every 100 patients treated for depression, CCBT would likely result in 21 more cases of clinically significant improvement compared with TAU, with an ICER of $3623 (95% CI, $2617-$5377) per case experiencing clinically significant improvement of at least 50% reduction in PHQ-9 score. The probability of CCBT being cost-effective was 95.1% at a WTP of $5000 per case of successful treatment response (Figure 3B).

## Discussion

This study presents a within-trial cost-effectiveness analysis of adding CCBT to TAU to treat depression in primary care. Over a follow-up period of 6-months posttreatment, CCBT was found to be associated with improvement in overall quality of life of patients, primarily attributed to improvement in mental health. The incremental cost per QALY was $37 295, which implies that, on average, CCBT is likely to improve health by 1 QALY at the additional cost of $37 295. For resource allocation decisions, health systems use specific threshold values to represent their willingness to pay (WTP) for an additional QALY. In the US, the commonly used WTP values range from $50 000 to $100 000 per QALY.^59,60^ Based on this, CCBT would be a cost-effective strategy for treating adult depression in primary care. Similarly, the incremental cost per additional case of reliable and clinically meaningful improvement, a measure useful for clinical decision-making, was $3623, which is comparable to previous estimates of CCBT for depression.^61,62^

Findings of our study align with systematic reviews of cost-effectiveness studies of computerized or internet-based interventions for depression in the adult population.^20,21,22,23^ Previous economic evaluations have compared guided, unguided or technically supported CCBT interventions with usual care in Australia,^63^ Canada,^64^ the Netherlands,^65,66^ Spain,^67^ Sweden,^68^ and the UK.^69,70^ Almost all studies found CCBT to be cost-effective compared with usual care in the adult population in relation to the WTP threshold used in the jurisdiction. Exceptions included Duarte et al (2017)^70^ conducted in the UK, which had treatment adherence and engagement of less than 20%, which reduced treatment effectiveness, and Holst et al (2018)^68^ conducted in Sweden, which had a small sample size of 65 patients (35 in TAU and 30 in the internet-based CBT) and did not achieve its recruitment target.^71^ None of the systematic reviews identified economic evaluations in the US; however, recently, Thase et al (2020)^17^ conducted a cost-effectiveness analysis of therapist-supported CCBT with the standard CBT over a 6-month period in the US and found it to be cost-effective. However, this study did not evaluate the incremental cost in relation to treatment response.

This study has a number of strengths. First, to our knowledge, this is one of the first studies to evaluate the cost-effectiveness of CCBT for depression in a sample with sufficient representation of socioeconomically marginalized populations, including those with limited access to the internet. With over half of study participants with income of less than $30 000 per year and three-quarters without college education, the findings of this study would allow decision-makers to understand the economic value of CCBT in a resource-diverse population. Second, previous studies of CCBT have provided limited material support, or even excluded participants who did not have access to a computer or an internet connection.^17^ These are known barriers to treatment enrollment, continuation and adherence, and addressing them can improve coverage of internet-based therapies. Our cost-effectiveness analysis incorporates the cost of loaner computers and internet connection for low-resource households. Third, we evaluated cost-effectiveness using both the QALY outcome, commonly used in the economic literature, and the treatment response outcome which is relevant from a clinical perspective. Reporting value for money for both outcomes is not common in the economic literature, as evidenced by previous systematic reviews.^20,21,22,23^ Another strength of the study relates to the relatively high treatment completion rate of 74.7% compared with the 58.3% reported in a recent meta-analysis.^19^

### Limitations

This study has limitations. First, participants in our trial had mild-to-moderate level of depression; therefore, the cost-effectiveness evidence should be interpreted for the same disease severity. Second, while the follow-up period of 6-months posttreatment is consistent with previous studies of CCBT, this study did not evaluate the long-term cost-effectiveness of CCBT, which would require strong modeling assumptions to estimate long-term outcomes and costs beyond the clinical trial. Third, the current study excluded nonintervention-related health services costs, as the trial did not collect these data. Previous studies indicate that nonintervention health service use is higher in the TAU group compared with the CCBT group,^61,64,65,66,68,69^ likely because CCBT may displace other services. This would potentially have the effect of lowering the incremental cost and the ICER, further strengthening the case for CCBT for depression. Additionally, because the current ICER already positions CCBT as a cost-effective strategy, including TAU costs would not change the recommendation for CCBT based on the threshold values used for decision-making. Third, the economic analysis was conducted from the perspective of the health system decision-maker, which does not include wider societal cost savings (eg, productivity gain) that may partly offset the CCBT intervention cost. The health system perspective is the most common and relevant viewpoint for resource allocation in the US and produces a conservative estimate of cost-effectiveness. Other limitations, including those related to the number of treatment sites and missing data, have been discussed in the previous study.^25^

## Conclusions

Given the population burden of depression and limited availability and accessibility to therapists, this economic evaluation’s results suggest that CCBT offers a viable alternative. This mode of treatment can substantially reduce clinician time with each patient, reduce wait time for treatment, overcome care-seeking stigma associated with in-person mental health care, provide a convenient and flexible treatment approach, and reduce cost for the health system and patients. Previous research had included insufficient numbers of persons with low income and/or low education and excluded those without internet access; however, our study has shown CCBT to be cost-effective in a sociodemographically diverse population. Given its low cost and comparable effectiveness, CCBT could be made available at large scale to improve access to treatment for depression across a wide socioeconomic spectrum.

## Notes

Supplement 1. **eTable 1.** Cumulative Costs and QALYs **eTable 2.** Base-Case and Extrapolation Using Longer Time Horizons for the Treatment Benefit

Supplement 2.
Data Sharing Statement
