# Supplementary material for: Cost-Effectiveness of Computer-Assisted Cognitive Behavioral Therapy for Depression Among Adults in Primary Care
Source: JAMA Netw Open. 2024 Nov 14;7(11):e2444599. doi: 10.1001/jamanetworkopen.2024.44599 (PMC11565263; doi:10.1001/jamanetworkopen.2024.44599)
Supplement: Supplement 1. — eTable 1. Cumulative Costs and QALYs eTable 2. Base-Case and Extrapolation Using Longer Time Horizons for the Treatment Benefit [file jamanetwopen-e2444599-s001.pdf]

Supplemental Online Content

Ali S, Alemu FW, Owen J, et al. Cost-effectiveness of computer-assisted cognitive behavioral therapy for depression among adults in primary care. *JAMA Netw Open.* 2024;7(11):e2444599. doi:10.1001/jamanetworkopen.2024.44599

eTable 1. Cumulative Costs and QALYs

eTable 2. Base-Case and Extrapolation Using Longer Time Horizons for the Treatment Benefit

This supplemental material has been provided by the authors to give readers additional information about their work.

© 2024 Ali S et al. *JAMA Network Open.*

eTable 1. Cumulative costs and QALYs

Time horizon	Group	Total Cost (95% CI)	Incremental Cost (95% CI)	Total QALY (95% CI)	Incremental QALY (95% CI)	ICER* (95% CI)
12-week	TAU	0	–	0.123 (0.122, 0.123)	–	–
	CCBT	714.64 (662.53, 766.75)	714.64 (662.53, 766.75)	0.128 (0.128, 0.129)	0.0059 (0.0022, 0.0097)	124,646.10 (88508.63, 179872.28)
3-month	TAU	0	–	0.270 (0.269, 0.270)	–	–
	CCBT	714.64 (662.53, 766.75)	714.64 (662.53, 766.75)	0.284 (0.283, 0.284)	0.0142 (0.0034, 0.0250)	53230.40 (34661.42, 91493.12)
6-month	TAU	0	–	0.416 (0.415, 0.416)	–	–
	CCBT	714.64 (662.53, 766.75)	714.64 (662.53, 766.75)	0.437 (0.436, 0.437)	0.0209 (0.003, 0.039)	37295.43 (22723.86, 66546.14)

* The values are based on 1000 multiple imputations and pooled using Rubin's rule.

© 2024 Ali S et al. *JAMA Network Open.*

eTable 2. Base-case and extrapolation using longer time horizons for the treatment benefit

Base-Case			
**Time horizon**	**Mean QALYs**	**Mean Cost ($)**	**ICER (95% CI)**
12-week	0.0059	714.64	124646.10 (88508.63, 179872.28)
3-month	0.0142	714.64	53230.40 (34661.42, 91493.12)
6-month	0.0209	714.64	37295.43 (22723.86, 66546.14)
**Extrapolation scenario 1: Assuming treatment benefit reduces over time**			
**Time horizon**	**Mean QALYs**	**Mean Cost ($)**	**ICER (95% CI)**
12-month	0.0389	714.64	19851.20 (11822.72, 35920.23)
24-month	0.0687	714.64	11541.41 (6526.72, 22784.19)
36-month	0.0900	714.64	8947.31 (4956.65, 18525.27)
48-month	0.1028	714.64	7899.44 (4320.28, 16652.41)
60-month	0.1070	714.64	7605.03 (4132.38, 16085.32)
**Extrapolation scenario 2: Assuming treatment benefit remains stable at the end of trial follow-up**			
**Time horizon**	**Mean QALYs**	**Mean Cost ($)**	**ICER (95% CI)**
12-month	0.0400	714.64	19341.12 (11499.22, 35223.39)
24-month	0.0783	714.64	10202.58 (5697.35, 20648.06)
36-month	0.1166	714.64	7020.52 (3764.04, 15175.45)
48-month	0.1549	714.64	5409.87 (2774.73, 12158.36)
60-month	0.1931	714.64	4495.60 (2207.93, 10080.23)

© 2024 Ali S et al. *JAMA Network Open.*
