# Supplementary material for: Cost-Effectiveness of Computer-Assisted Cognitive Behavioral Therapy for Depression Among Adults in Primary Care
Source: JAMA Netw Open. 2024 Nov 14;7(11):e2444599. doi: 10.1001/jamanetworkopen.2024.44599 (PMC11565263; doi:10.1001/jamanetworkopen.2024.44599)
Supplement: Supplement 2. — Data Sharing Statement [file jamanetwopen-e2444599-s002.pdf]

Data Sharing Statement

Ali. Cost-Effectiveness of Computer-Assisted Cognitive Behavioral Therapy for Depression Among Adults in Primary Care. *JAMA Netw Open*. Published November 13, 2024. doi:10.1001/jamanetworkopen.2024.44599

Data
Data available: No
